# Assessment of the Quality and Reliability of YouTube as an Information Source for Transforaminal Interbody Fusion

**DOI:** 10.7759/cureus.50210

**Published:** 2023-12-09

**Authors:** Yusuf Bayram, Ertuğrul Pınar

**Affiliations:** 1 Orthopedics and Traumatology, Hisar Intercontinental Hospital, Istanbul, TUR; 2 Neurological Surgery, Special Pendik Yuzyil Hospital, Istanbul, TUR

**Keywords:** gqs score, videos, fusion, interbody, transforaminal, lumbar, quality, reliability, tlif, youtube

## Abstract

Background: This study aims to assess the quality and reliability of the information for patients from YouTube videos on transforaminal interbody fusion (TLIF).

Material and methods: One hundred videos were listed by inputting “TLIF,” “TLIF surgery,” and “transforaminal interbody fusion” in the YouTube search engine. The top 50 most popular videos based on video power index (VPI), view ratio, and exclusion criteria were selected for review. One orthopedic consultant surgeon and one neurosurgeon consultant analyzed the videos together. The modified DISCERN score, the Global Quality Score (GQS), the Journal of the American Medical Association (JAMA) score, and a novel interbody fusion score were used to evaluate videos. Data of video length, view count, number of likes and dislikes, like ratio (like x 100/(like+dislike)), video source, and comment rate were collected.

Results: The quality of the videos could have been better according to all scoring systems, regardless of the video source. The scores of the videos published by patients and commercials were significantly lower than those of physicians and allied professionals (p <0.05). VPI and view ratios were similar in all sources.

Conclusion: The study demonstrates that YouTube videos providing information related to TLIF surgery are available and accessed by the public. The results of this study would suggest that YouTube is not currently an appropriate source of information on TLIF surgery for patients. Most of the YouTube videos about TLIF surgery contain information about the surgical technique and have limited information about the post-operative condition of the patients.

## Introduction

Lumbar interbody fusion is used to treat lumbar spinal disorders, such as spinal stenosis, recurrent disc herniation, lumbar and lumbosacral instability, spondylolisthesis, and pseudoarthrosis [1]. Lumbar interbody fusion contributes to the restoration of intervertebral disc distance, foraminal height, and proper sagittal alignment of the spine. Surgery aims to provide neurological recovery and stability in the spine, stop the progression of slippage, and increase the patient's quality of life by reducing pain [1].

Transforaminal lumbar interbody fusion (TLIF), posterior lumbar interbody fusion (PLIF), lateral lumbar interbody fusion (LLIF), oblique lumbar interbody fusion/anterior to psoas (OLIF/ATP), and anterior lumbar interbody fusion (ALIF) are commonly used surgical options for lumbar spine interbody fusion [2]. Transforaminal lumbar interbody fusion (TLIF) is a modification of the posterior lumbar interbody fusion technique (PLIF) and was first applied by Harms and Rolinger in 1982 [3]. TLIF is gaining popularity with its high fusion rate, good clinical results, and low complication rates [4,5].

Although patients are informed about the procedure and its complications by their doctors, they also research the details of the procedure, the healing process, and what kind of problems they will encounter after surgery from different sources, mostly on the Internet [6-8].

Video resources are also frequently used to watch the surgeries to be performed, to see different patient experiences, and to see the evaluations of different doctors and institutions [9]. YouTube is one of the most basic video-sharing sites where it is easy to access and most information can be obtained [10-12]. Since the content of the videos cannot be checked by the experts on the subject. At the same time, they are being uploaded; there are doubts about the accuracy and reliability of the information. 

The current study aims to assess the quality and reliability of the information in YouTube videos on transforaminal interbody fusion.

## Materials and methods

A search was done on the YouTube™ (https://www.youtube.com) channel on March 9, 2022, with the keywords “TLIF,” “TLIF surgery,” and “Transforaminal interbody fusion"-the top 100 videos listed. YouTube's popularity ranking was used when ranking the videos, and the 100 most popular videos were examined. The videos after the top 100 were not included because of very low viewing rates. Less than 1000 views (n=30), shorter than 90 seconds (n=8), non-English videos (n=2), repetitive videos (n=1), and silent videos (n=9) were determined as exclusion criteria. In order to obtain more objective results, one orthopedic surgeon and one neurosurgeon (YB and EP) evaluated and scored all the videos separately in a double-blind manner, and then the average of the obtained values ​​was taken.

The video title, time since upload (months), total view count, daily view count, video length, number of likes and dislikes, like ratio (like × 100/(like + dislike)), number of comments, comment ratio (comment/year), and video source were noted for each video [13-15]. YouTube data valid from the date of upload until September 22, 2022, was evaluated for all videos.

The video power index (VPI) and view ratio were used to assess the videos’ popularity. Erdem et al. described VPI, which was calculated using (like ratio × view ratio/100) [15]. The view ratio was calculated as (number of views/time since upload) [15]. 

The videos were evaluated according to a Global Quality Score (GQS), modified DISCERN scores, the Journal of the American Medical Association (JAMA) score, and a novel interbody fusion score. Modified DISCERN scores (Table 1) obtained by Singh et al. evaluate health information in YouTube videos [16-19]. This five-point global score evaluates the reliability of data with five questions [20].

**Table 1:** Modified DISCERN scale (yes: 1 for each question, no: 0 points).

Is the video clear, concise and understandable?
Is it obtained from valid sources? (i.e., publication cited, speaker is spine surgeon)
Is the information presented balanced and unbiased?
Are additional sources of information listed for patient reference?
Are areas of uncertainty mentioned?

The JAMA benchmark evaluates the quality of online information based on four criteria: authorship, attribution, disclosure, and currency. Each criterion corresponds to one point, and four points indicate high quality [20-23]. GQS evaluated the educational quality of the videos. GQS is scored from one to five; one indicates low quality, and five indicates high quality [24-26].

Lumbar interbody fusion scoring (Table 2) was performed using a previously described interbody scoring system. Reliability and comprehensiveness scores were given out of five and 12 points, respectively. In scoring, three groups were created by looking at comprehensiveness: good (9-12), average (4-8), and poor (0-3) [27].

**Table 2:** Interbody fusion scoring.

Item	Point
Section A: Preoperative education	
Discussion of nonoperative options	1
Discussion on the concept of spinal fusion	1
Discussion of indications for surgery	1
Preoperative preparation	1
Section B: Surgical	
Discussion of patient positioning and type of anesthesia	1
Discussion of surgical procedure and techniques	1
Discussion of instrumentation used for spinal fusion	1
Discussion of open vs minimally invasive vs endoscopic	1
Section C: Postsurgical	
Discussion of post-operative mobilization and/or physiotherapy and rehabilitation	1
Discussion on functional outcome (improved mobility, pain, quality of life, and so forth)	1
Discussion on possible complications including but not limited to (infection, cerebrospinal fluid leak, pseudoarthrosis, nerve or sac injury, implant failure, venous thromboembolism, resurgery)	0.5 Each, for a maximum of 2 points

The source of the videos was classified into four categories: physicians, other medical professionals (such as physical therapists or alternative medical providers), commercial, and patients. The videos were also evaluated according to whether they were animated or non-animated.

Statistical analysis

In our study, the distribution of continuous variables was evaluated with the Shapiro-Wilk test, skewness and kurtosis values, and histogram graphics. The kurtosis statistics were above 1.5 for the continuous variables that we tested. Histogram graphics did not show a normal distribution. Then the appropriate tests were administered. Since continuous variables do not show a normal distribution, median (IQR, interquartile range) and categorical variables are shown as frequency (percentage).

The relationship between two continuous variables was evaluated with the Spearman correlation test. The correlation level was classified as <0.2 (very weak), 0.2-0.4 (weak), 0.4-0.6 (moderate), 0.6-0.8 (high), >0.8 (very high). The chi-square test was used for categorical variables, and Kruskal-Wallis and Mann-Whitney U tests were used for continuous variables in comparisons between groups. The statistical significance level was accepted as <0.05.

## Results

One hundred videos searched as “TLIF,” “TLIF surgery,” and “transforaminal interbody fusion” were listed. Fifty TLIF surgery videos were evaluated in our study that met the criteria. Descriptive statistics of the data are presented in Table 3.

**Table 3:** Descriptives of the results. GQS: Global Quality Score, JAMA: Journal of the American Medical Association.

	Median	Minimum	Maximum	IQR	Mean	Standard deviation
GQS	2.00	1.00	4.00	1.00	1.82	0.87
JAMA	1.00	0.00	3.00	0.00	1.08	0.80
Modified DISCERN score	4.00	0.00	10.00	1.00	4.18	2.33
Interbody fusion score	1.00	0.00	5.00	2.00	1.42	1.11
Video length/min	6.45	1.10	223.10	9.23	18.90	40.37
View count	10435.50	1548.00	1581639.00	32641.00	67249.40	226922.96
Time since upload/months	62.50	14.00	212.00	84.00	72.92	47.71
View ratio	7.97	0.42	365.19	19.13	27.51	61.99
Like	69.50	1.00	3500.00	130.00	265.18	683.94
Dislike	0.00	0.00	0.00	0.00	0.00	0.00
Like ratio	100.00	100.00	100.00	100.00	100.00	0.00
Number of comment	2.00	0.00	573.00	14.00	29.66	93.60
Comments/year	0.62	0.00	286.50	3.08	9.16	40.68
VPI (like ratio × view ratio/100)	7.97	0.42	365.19	19.13	27.51	61.99

The total number of views of 50 videos is 3,362,470 (range: 1548-1581639), with an average of 67,249 views per video. Only 0.4% of the viewers liked the videos, but no one disliked the videos. That's why the video-like ratio is set to 100%. The mean time since upload was 73 months (SD=48), and the mean view ratio was 27.51 (SD=61.99). The mean number of comments for each video was 30 (SD=94), and 9.16 comments were made in one year per video. We used four different scoring systems to show the quality and reliability of the videos more accurately.

According to the lumbar interbody fusion scoring, four videos (8%) were good, 22 (44%) were average, and 24 (48%) were poor. According to modified DISCERN scoring, one (2%) video was excellent, three (6%) were good, three (6%) were average, eight (16%) were poor, and 35 (70%) were very poor. GQS showed zero (0%) videos were excellent, four (8%) were good, three (6%) were fair, 23 (46%) were poor, and 20 (40%) were very poor.

The GQS score positively correlated with the TLIF score, JAMA, and modified DISCERN (rho, 0.791, p < 0.001; rho, 0.493, p < 0.001; rho, 0.651, p < 0.001, respectively). Among 50 videos, 38 (76%) were non-animated, and 12 (24%) were animated. Although the quality scores, VPI and view ratios of non-anime videos were higher, this was not statistically significant (p > 0.05).

The source of videos was a physician in 38 (76%) videos, other medical professionals in two (4%), a commercial in eight (16%), and a patient in two (4%). The quality of the videos was poor according to all scoring systems, regardless of the video source. The scores of the videos published by patients and commercials were significantly lower than those of physicians and other medical professionals (p < 0.05). VPI and view ratios were similar in all sources. 

The quality of information on YouTube regarding TLIF surgery could have been better. The videos were found to lack information on the post-operative period, indications and risk factors for surgery. Data from this study should guide spine surgeons as they develop and post videos to YouTube to create informative and comprehensive videos.

## Discussion

In recent years, YouTube has become increasingly popular, not only for spinal procedures but also for patients' access to information in other fields of medicine [28]. The main finding of our study was that the reliability and accuracy of YouTube videos about TLIF surgery were low compared to all scores.

The mean GQS, modified DISCERN, JAMA score, and TLIF score were 2, 1, 1, and 4, respectively, and these low scores reflected insufficient quality. These low-scoring results are consistent with previous studies [20,29]. Erdem et al. evaluated the quality of videos on YouTube about kyphosis; the videos had a mean JAMA score of 1.36 (range: 1 to 4), GQS score of 1.68 (range: 1 to 5), and kyphosis-specific score of 3.02 (range: 0 to 32) [15].

A study assessing the quality of cervical disc replacement videos showed that overall, video quality and educational content were low [30]. Also, another systematic review study about lumbar spinal fusion videos’ reliability according to the DISCERN scoring criteria and comprehensiveness using their novel scoring system showed that most videos (75.7%) were poor in comprehensiveness [27]. Similar to other studies, our study showed that the video source is an important parameter in evaluating video quality [14]. However, these videos still needed to be more safe and sufficient information.

Although we saw that the quality of non-anime videos was higher than that of anime videos, we could not prove this statistically. Celik et al. reported that animated videos had higher VPI scores than non-animated videos [14]. However, commercial websites usually upload animated videos so that the quality scores could have been better [14]. We didn’t find a correlation between the duration of videos and quality. It was clear that the videos mostly contained technical topics that could be used in surgical training on how surgery is performed and information that patients would need help understanding.

In the study, although the scores of the videos were low, in general, the quality and reliability of the videos published by the physicians were higher. In the videos, it was seen that indications, complications, disadvantages of surgery, and post-operative rehabilitation were not sufficiently mentioned. Apart from providing written information about the surgery, patients should be shown an online source to obtain the correct information.

Surgical videos uploaded to YouTube should be supervised, experienced centers should check the reliability of the information, and it should be stated to whom the information is addressed. Videos that do not meet certain criteria should be warned while viewing by patients.

Limitations

Our study had certain limitations. We only evaluated videos on YouTube and in English. This study only evaluates the surgery videos on YouTube and does not provide information about different internet platforms. The doctor does the scoring of the videos and does not express the opinion of the patients. Scoring was done by only two doctors. This study had strengths as well as limitations.

This study focuses on a specific and popular topic in spine surgery. Most of the videos on YouTube were evaluated by both orthopedists and neurosurgeons dealing with spine surgery using multiple scoring systems. This is the first objective study evaluating the TLIF surgery videos.

## Conclusions

The study demonstrates that YouTube videos providing information related to TLIF surgery are available and accessed by the public. Low scores were obtained in all GQS, JAMA scoring, modified DISCERN scoring, and interbody fusion scoring. No significant effect of parameters such as viewing rate, number of likes, and video duration on the scoring was observed. The most notable finding on the scores was the video source. The results of this study would suggest that YouTube is not currently an appropriate source of information on TLIF surgery for patients. Because of this, there is a need for different platforms where the videos are scrutinized and regulated. Moreover, studies that include different platforms where more videos are evaluated by more authors are required for the most reliable and quality information.
